# Detection of Lungs Status Using Morphological Complexities of Respiratory Sounds

**DOI:** 10.1155/2014/182938

**Published:** 2014-02-06

**Authors:** Ashok Mondal, Parthasarathi Bhattacharya, Goutam Saha

**Affiliations:** ^1^Department of Electronics and Electrical Communication Engineering, Indian Institute of Technology, Kharagpur, Kharagpur 721 302, India; ^2^Institute of Pulmocare and Research, Kolkata, Kolkata 700 064, India

## Abstract

Traditionally, the clinical diagnosis of a respiratory disease is made from a careful clinical examination including chest auscultation. Objective analysis and automatic interpretation of the lung sound based on its physical characters are strongly warranted to assist clinical practice. In this paper, a new method is proposed to distinguish between the normal and the abnormal subjects using the morphological complexities of the lung sound signals. The morphological embedded complexities used in these experiments have been calculated in terms of texture information (lacunarity), irregularity index (sample entropy), third order moment (skewness), and fourth order moment (Kurtosis). These features are extracted from a mixed data set of 10 normal and 20 abnormal subjects and are analyzed using two different classifiers: extreme learning machine (ELM) and support vector machine (SVM) network. The results are obtained using 5-fold cross-validation. The performance of the proposed method is compared with a wavelet analysis based method. The developed algorithm gives a better accuracy of 92.86% and sensitivity of 86.30% and specificity of 86.90% for a composite feature vector of four morphological indices.

## 1. Introduction

The audio information of respiratory signals is used to find out the pulmonary dysfunctions. The diagnostic status of the respiratory system can be assessed by interpreting the audible characteristics of lung sound signals in terms of varying amplitude, intensity, and tone quality or modal frequencies. Physicians examine the lung disease in two ways: one is noninvasive process which includes auscultation, pulmonary function test, respiratory inductance plethysmograph, and phonopneumography technique and the other is invasive approach such as chest X-ray or roentgenogram and computerized tomography (CT) scan, and so forth. The invasive diagnostic procedures are expensive, time consuming, and harmful as in case of X-ray repetition. One of the popular noninvasive approaches is auscultation, a stethoscope device based technique, started after the invention of stethoscope by French physician Laennec in 1962 [1]. It is a most simple and inexpensive diagnostic tool and widely used by medical practitioners. However, this cost effective and easily handling appliance is unable to remove interventions produced from the surroundings organs, namely, cardiac sound source and also form the clinical environment that leads to misdiagnosis of the respiratory diseases.

The nonlinear and nonstationary properties of lung sound signal make it difficult to diagnose the lungs status using only the temporal or spectral characteristics of the respiratory sounds. The lung sound (LS) shows a complex dynamics because of the involvement of transmission path filtering effect, attenuation, and its production mechanism which is unstable. The pathological status of the lungs signifies a morphological deviation of the normal breath sound. The pattern complexities of abnormal LS are higher than that of the normal LS because of the occurrence of auxiliary signals in case of unhealthy lungs. The doctors take help of different clinical devices, namely, modern electronic stethoscope, CT scan, bronchoscopy, and so forth, to capture the various distinctive parameters of the normal as well as abnormal states of the lungs. The accuracy of diagnosis with these diagnostic tools depends on the experience and knowledge of the physicians and also on the cooperation of the patients.

With the advances of computer technologies, statistical signal processing, artificial intelligence, and pattern recognition algorithms, lung sound analysis is commenced in an automated manner. The computer aided or microprocessor based automated tools offer several facilities in terms of high speed, large storage capacity and avoid the manual hazards. The important intervening step of automated lung sound analysis is the extraction of authentic features that are inherently correlated with the lungs conditions. The final stage of pattern recognition based lung sound analysis system is decision making about the underlying disease, if any. The aim of feature extraction procedure is to identify the relevant distinct parameters of the LS signals and to arrange them in vector form that serves as an input during classification. Researchers have developed several feature extraction techniques to form the feature vectors based on parametric and nonparametric methods. In parametric technique models the LS signal based on a priori knowledge is in contrast with nonparametric method which characterizes LS signal using a set of basis functions [2]. Many studies have been done in classifying the lung dysfunctions which suggest a variety of methods based on time, frequency, and time-frequency domain analysis [2–10].

The first initiative for lung sounds analysis was taken by Forgacs et al. in the late 1960s [11]. After that a number of research works were published in this field. In 1973, Murphy Jr. and Sorensen described a spectral based technique for wheeze sounds analysis [12] and later on Murphy Jr. et al. introduced a waveform analysis of crackles sounds [13]. Dosani and Kraman presented a technique called phonopneumography in 1983 [14] for measuring the intensity variation of normal lung sounds. In 1991, Tinkelman et al. [15] suggested a computer digitized phonopneumography technique to detect the airway obstruction in child patients. A classification method has been developed by Cohen and Landsberg to distinguish between normal and abnormal breath sounds by computing the Mahalanobis distance between the known and unknown classes using their mean feature vectors and covariance matrix of known data [5]. This is a semiautomated system because breath cycles are selected manually and inapplicable for adventitious sounds. Sankur et al. proposed a technique to discriminate pathological from normal subjects using autoregressive coefficients and k-nearest neighbour and quadratic classifier in [2]. Gavriely et al. [4] have analyzed normal lung sounds and characterized these sounds by defined some parameters such as amplitude, frequency, and regression lines slopes corresponding to high and low frequency segments. They have suggested that these parameters are useful for discrimination between normal and pathological sounds. A neural network based classification technique has been proposed by Yeginer et al. [6] for distinguishing between normal and abnormal subjects using the subphase features like autoregressive coefficients, prediction error, and ratio of expiration and inspiration duration. The correct classification performance for a small database is between 70 and 80%. The efficiency of this procedure degrades due to improper selection of model order. Zheng et al. [7] have introduced an algorithm to differentiate between normal and abnormal lung sounds using time and frequency domain features combined with a stochastic classification procedure. Matsunaga et al. have presented a maximum likelihood approach based method in distinguishing the adventitious and normal pulmonary sounds [8]. This classification technique uses two types of acoustic modeling methods: one is hidden Markov model (HMM) for detection of adventitious sounds and another one is microphone dependent model to identify the normal sounds. This is a semiautomated processes because the acoustic segments are labeled manually and its performance degrades for noisy data.

Marshall and Boussakta [9] have developed a classification technique to classify the normal and crackles sounds using the wavelet features and cross-correlation mathematical tool. A wavelet based classification approach has been developed by Kandaswamy et al. [10] for discriminating six different types of pulmonary sounds by using statistical features of the wavelet detail coefficients (D1 to D7), produced by Daubechies wavelet of order 8 and artificial neural network. Another wavelet based analysis has been studied by Sello et al. [3] to separate healthy subject from pathological subject by characterizing the frequency power distribution of the wavelet coefficients generated by Morlet wavelet from respiratory sounds.

The difficulties are faced by researchers in analysis of respiratory sounds that are the interference of lung sounds with heart sounds, appearance of different pathological sounds in similar forms, and also the unavailability of the sophisticated instrumentation for processing information embedded in the lung sounds. The focus of the study is to develop a new method for better analysis of lung sounds by exploring the statistical approaches and digital signal processing knowledge combined with pattern recognition algorithms. In this paper, a new technique is proposed to detect the lungs status, normal and abnormal, using the structural complexities of LS signals. The structural behavioral of the LS signal is parameterized with a number of distinct features such as sample entropy, lacunarity, skewness, and kurtosis. A twenty-four-dimensional feature vector is formed using these parameters. The ELM and SVM networks are used to evaluate the efficiency of the developed technique. The proposed technique gives better performance than the baseline method [3].

The rest of this paper is organized as follows. Theoretical background information on ELM and SVM classifiers is described in Section 2. The methodology to distinguish between normal and abnormal status of the lungs is discussed in detail in Section 3 and the database and implementation platform that are employed in the work are discussed in Section 4.  Section 5 depicts the experimental results and discusses the efficiency of the method and conclusion is given in Section 6.

## 2. Theoretical Background

### 2.1. Extreme Learning Machine (ELM)

In 2006, Huang et al. proposed a high speed and simple learning algorithm to remove the drawbacks of conventional learning algorithms for a single layer feed forward network (SLFN) [16]. This learning technique is named as extreme learning machine (ELM) which is thousands times faster than conventional learning approaches because it avoids the adjustment of hidden layer parameters (weights and biases) during training by choosing them randomly. The ELM algorithm trains a SLFN through the three steps which are given in the next.

Consider an activation function *g*(*y*) which is infinitely differentiable, hidden nodes $\tilde{N}$, and a training set *S* = {(*y*_*p*_, *d*_*p*_), *p* = 1,2,…, *N*}; here, *d*_*p*_ ∈ *R* ^*m*^ is the output response for the input sample *y*_*p*_ ∈ *R* ^*n*^.

**Step 1**

Assign hidden layer biases *b*_*j*_ and input weights *w*_*j*_ randomly according to any continuous probability density function, $j=1,2,\ldots ,\tilde{N}$.

**Step 2**

Compute the output matrix **M** of the hidden layer.

**Step 3**

Compute the output weights $\overset{ˇ}{\beta }=M^{†}D$, where **D** = [*d*_1_,…, *d*_*N*_]^*T*^ and **M** ^†^ is the Moore-Penrose generalized inverse of the hidden layer matrix **H** [17, 18].

**Figure 1:**
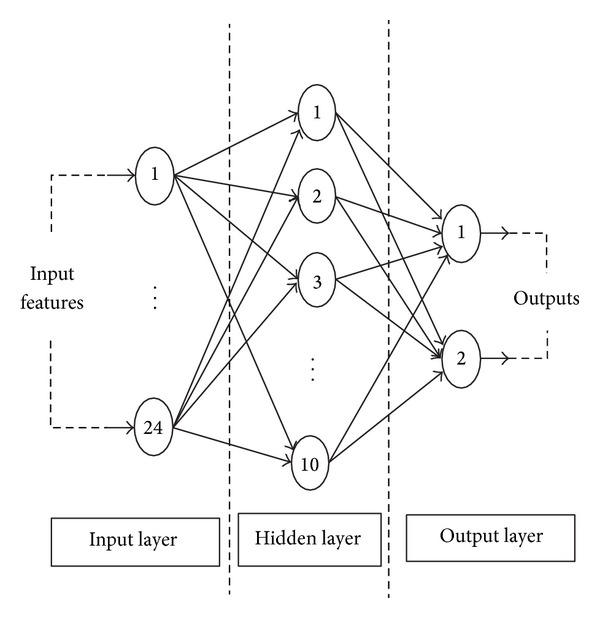
Structure of ELM network.

However, the classification accuracy of the ELM network depends on the number of hidden nodes and the selection of the activation functions. In this work we have chosen radial basis activation function and an ELM network whose hidden layer consists of 10 hidden nodes because it gives better accuracy than the other combinations. The structure of an ELM network is shown in Figure 1. This network is used in our experiments.

### 2.2. Support Vector Machine (SVM)

The support vector machine (SVM) network was proposed by Cortes and Vapnik in 1995 as an alternative tool of multilayer feedforward neural network [19]. SVMs are used to solve the classification and regression problems. The SVM classifies different patterns through the two steps: (1) first the training data are mapped to a feature space of high dimension using a nonlinear kernel function and (2) after that an optimal hyperplane is constructed using the method of Lagrange multipliers in order to separate the individual classes. The hyperplane is used to distinguish two linearly separable classes as given by

(1) $$\begin{array}{c} d_{j}\left(\omega^{T}f\left(y_{j}\right)+b\right)\geq 1,\;\text{for}\;\;j=1,2,\ldots ,L, \end{array}$$

where *y*_*j*_ ∈ **R** ^*n*^ is *j*th input pattern and *d*_*j*_ ∈ {−1,1} is the corresponding output pattern or target for a training dataset {*y*_*j*_, *d*_*j*_}_*j*=1_^*L*^. *f*(·) is a nonlinear mapping function.

The decision surface of (1) is modified by introducing a nonnegative slack variable *ξ* in order to separate two nonlinearly separable classes as represented by

(2) $$\begin{array}{c} d_{j}\left[\omega^{T}f\left(y_{j}\right)+b\right]\geq 1-\xi_{j},\;\text{for}\;\;j=1,2\ldots ,L. \end{array}$$

An optimal hyperplane can be obtained by minimizing the function *F*(*ω*, *ξ*) with respect to *ω* and *ξ*_*j*_ and it is expressed by

(3) $$\begin{array}{c} F\left(\omega ,\xi \right)=\frac{1}{2}\omega^{T}\omega +\Gamma \overset{L}{\underset{j=1}{\sum }}\xi_{j}, \end{array}$$

where Γ is the reciprocal of a regularization parameter and it controls the tradeoff between complexity of the machine and the number of nonseparable points [20].

To construct a decision function *ϕ*(*y*) (4) for an SVM classifier, it is required to maximize the objective function *Q*_*f*_(*α*) with respect to Lagrange multipliers {*α*_*j*_}_*j*=1_^*L*^, subject to the two constraints expressed by (6):

(4) $$\begin{array}{c} \phi \left(y\right)=\mathrm{sign}\left(\overset{L}{\underset{j=1}{\sum }}\alpha_{j}d_{j}K_{f}\left(y,y_{j}\right)+b\right), \end{array}$$

(5) $$\begin{array}{c} Q_{f}\left(\alpha \right)=\overset{L}{\underset{j=1}{\sum }}\alpha_{j}-\frac{1}{2}\overset{L}{\underset{j=1}{\sum }}\;\overset{L}{\underset{i=1}{\sum }}\alpha_{j}\alpha_{i}K_{f}\left(y_{j},y_{i}\right)d_{j}d_{i} \end{array}$$

subject to

(6) $$\begin{array}{c} \overset{L}{\underset{j=1}{\sum }}d_{j}\alpha_{j}=0,\;0\leq \alpha_{j}\leq \Gamma ,\;\;j=1,2,\ldots ,L. \end{array}$$

The kernel function *K*_*f*_(*y*, *y*_*j*_) must satisfy the Mercer's condition.

## 3. Methodology

### 3.1. Enhancement of Lung Sound Signals

**Figure 2:**
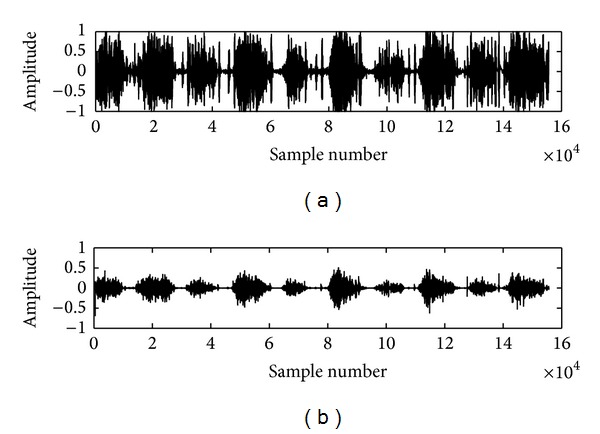
(a) It depicts the waveform of a noisy lung sound signal. (b) It shows the corresponding differentiation output of the nosy normal LS.

**Figure 3:**
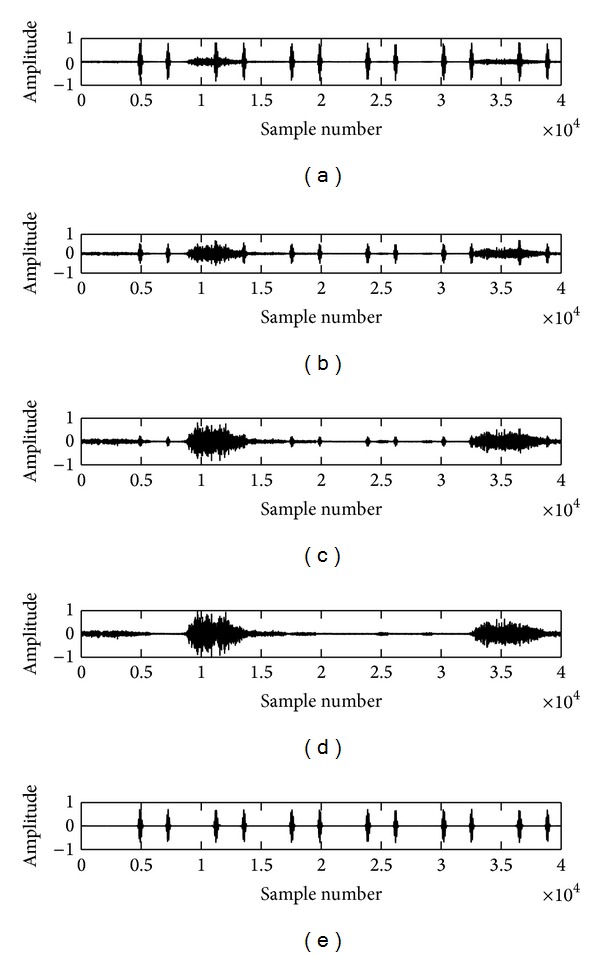
Graphical results of the EMD based HS removal technique (proposed by us). (a) It shows the waveform of a mixed LS signal (20% LS + 80% HS). (b) It shows the waveform of a mixed LS signal (50% LS + 50% HS). (c) It shows the waveform of a mixed LS signal (80% LS + 20% HS), (d) Reconstructed LS signal, (e) Residual HS signal.

The recorded lung sounds are contaminated with environmental noise, manmade artifacts, data recording and processing instruments' disturbances, and heart sound interference which leads to an incorrect detection of the lungs conditions. In this work, we have reduced the surrounding noise by recording the lung sounds in a quite environment and manmade artifacts are suppressed by placing the stethoscope in a proper way over the recording positions of the subjects. The instrumental disturbances are removed with a first order differentiation algorithm [21] and the heart sound (HS) noise is eliminated using a novel algorithm based on empirical mode decomposition (EMD) technique developed by us and filled an Indian patent (Ref. number 515/KOL/2011) and the work has been published in [22]. The results of the first order differentiation algorithm and EMD based technique are depicted in Figures 2 and 3, respectively. The enhanced lung sound signals are used in the next step to extract the embedded features for differentiating the normalcy and abnormality.

### 3.2. Respiratory Cycle Calculation

It is seen that inspiration and expiration phase of a lung sound cycle carries significant information regarding the lungs conditions. Hence, one complete breathing cycle is required to diagnose the lungs' status using morphological complexities of the respiratory sound signals. In this study, we calculate one complete respiratory cycle using a new algorithm developed by us based on Hilbert transform (HT) [23] and published in [24]. The algorithm can be summarized as follows.

**Step 1**

Calculate envelope ${\tilde{H}}_{e}(t)$ of the test signal *y*(*t*) using HT

(7) $$\begin{array}{c} {\tilde{H}}_{e}\left(t\right)={\left[y{\left(t\right)}^{2}+\hat{H}{\left(x\left(t\right)\right)}^{2}\right]}^{1/2}, \end{array}$$

where $\hat{H}(\cdot )$ is the Hilbert opertor.

**Step 2**

Smoothing of envelope signal ${\tilde{H}}_{e}(t)$ is done by a finite impulse response (FIR) Butterworth filter of cut-off frequency 5 Hz. This operation minimizes the fast vibration of ${\tilde{H}}_{e}(t)$.

**Step 3**

The transition points *t*_*p*_*j*=1,2,…,*n*__ of breathing phases are determined using the first order derivative of the smoothen envelope signal ${\tilde{H}}_{e}^{s}(t)$ according to the following rule:

(8) $$\begin{array}{c} {\left[\frac{d(H_{e}^{s}(t))}{dx}\right]|}_{t=t_{p_{j}}-1}<0,\\ {\left[\frac{d(H_{e}^{s}(t))}{dx}\right]|}_{t=t_{p_{j}}}=0,\\ {\left[\frac{d(H_{e}^{s}(t))}{dx}\right]|}_{t=t_{p_{j}}+1}>0. \end{array}$$

**Figure 4:**
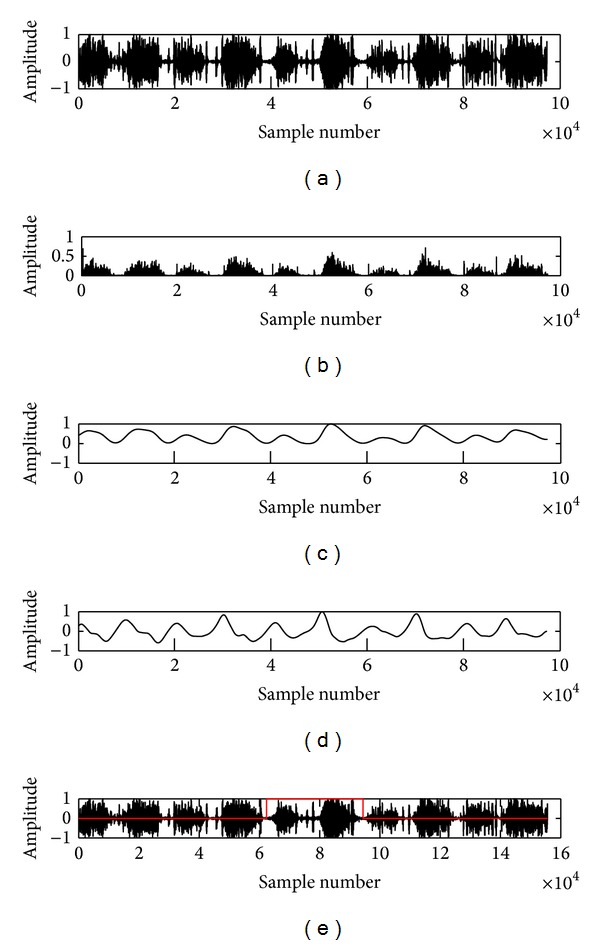
(a) depicts the waveform of a normal LS signal. (b) It shows the Hilbert envelope and (c) shows the smoothen envelope. (d) First order derivative of the smoothen envelope curve. (e) The red line defined a respiratory cycle estimated by the distance between two consecutive phases.

The duration of inspiration or expiration phase is calculated by measuring the distance between two consecutive minima points *t*_*p*_*j*__. The cycle duration *C* ^*d*^ of lung sound signal is determined by Algorithm 1. The results of the cycle detection algorithm are shown in Figure 4.

### 3.3. Characterizing Parameters of Lung Sounds and Feature Extraction Techniques

#### 3.3.1. Essential Parameters of Lung Sounds to Capture the Pattern Changes

**Figure 5:**
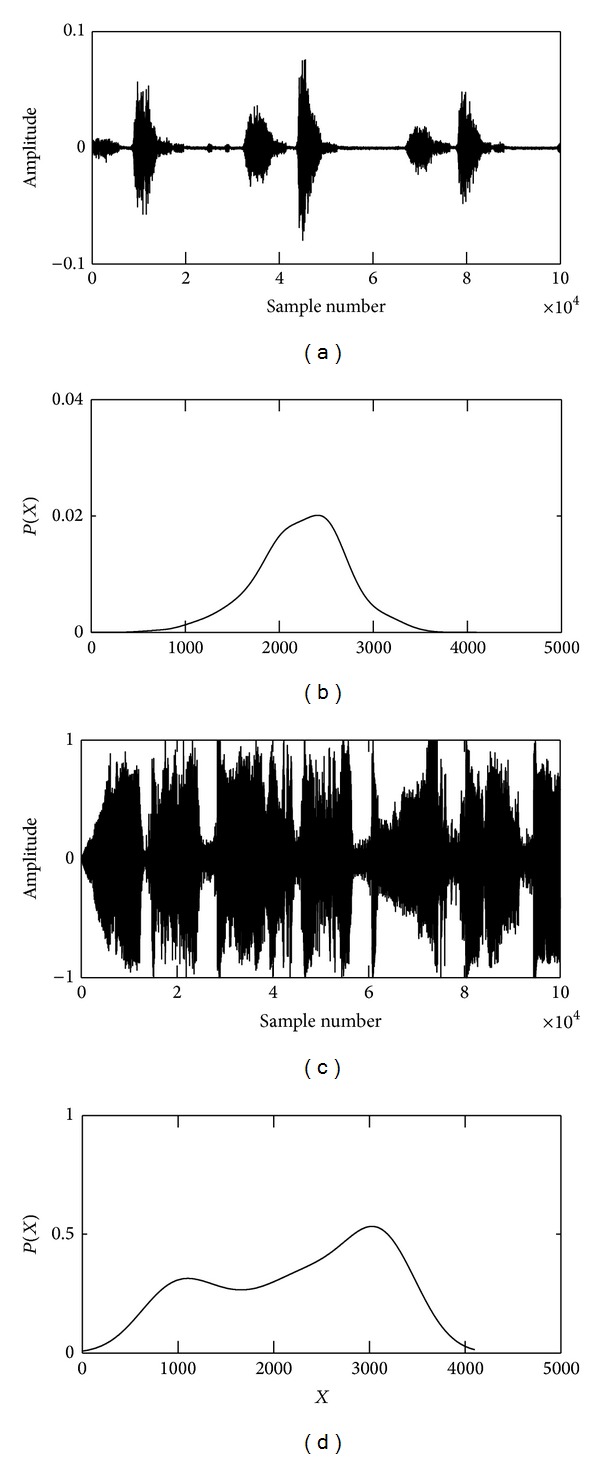
(a) and (c) display the normal and abnormal lung sound waveforms. (b) and (d) show the probability distribution functions for normal and abnormal cases.

One of the most important steps in respiratory sounds analysis is to identify the relevant inherent properties of the lung sound signals. These attributes are useful for modeling the healthy and unhealthy conditions of the lungs. The respiratory sounds are complex in nature because of the randomized vibration of the air ways walls and turbulent flow of gases through the respiratory system. The dynamical complexities of the abnormal lung sound are higher than that of the normal lung sound because of the presence of auxiliary sound in abnormal cases. Hence, morphological patterns of the abnormal lung sounds deviate from that of the normal sounds in a certain degree of alignment. The temporal domain features are not relevant in diagnosing of respiratory diseases because of the equivalent resemble for the both cases of normal and abnormal patients. On the other hand, the spectral domain characteristics of the lung sounds do not meet the requirements of pattern recognition due to the nonstationary behavior of the signals. The respiratory signals show non-stationarity characteristic because of the change in lung volume during the breathing process [25]. In this work, the statistical domain features are explored to measure the morphological complexities of the lung sound signals. The feature sets consist of four statistical parameters: kurtosis (*χ*), skewness (*ξ*), lacunarity (*ζ*), and sample entropy (*α*). Among these features kurtosis and skewness parameters can measure the flatness and asymmetric distribution of the probability density functions (PDFs) for normal and abnormal lung sound signals as shown in Figures 5(b) and 5(d), respectively. It is seen from Figure 5 that the flatness and asymmetric distribution of the abnormal LS are higher compared to normal lung sound. Hence, the kurtosis and skewness values for abnormal LS must be higher than that of the normal LS. The lacunarity (*ζ*) parameter measures the texture or heterogeneity information of any objects which may be fractal or nonfractal [26]. The texture of normal LS is different from that of the abnormal LS because of their different genesis mechanism. Hence, the texture information of the normal and pathological respiratory signals can be measured through the lacunarity index. The abnormal LS is more heterogeneous and correspondingly gives a higher lacunarity value than the normal LS. The sample entropy (*α*) is a statistical parameter which can measure the complexity or irregularity in a signal [27]. The sample entropy value increases with the irregularity property of the signals and vice versa. The abnormal LS is irregular or more complex in nature than that of the normal lung sound signal due to the unstable condition of the respiratory system associated with the disease severity. Hence, abnormal lung sound gives a higher sample entropy value over the normal one and we have verified it in our preliminary work which has been published in [24]. The complexity of the LS alters with the pathological conditions of the lungs. The morphological complexity of the pathological signals is different from that of the normal lung sound signals. These statistical parameters (*χ*, *ξ*, *ζ*, *α*) can significantly quantify the morphological changes in pathological and normal respiratory signals. Hence, these features may be useful in discriminating the normal and abnormal conditions of the lungs.

#### 3.3.2. Feature Extraction Techniques

In this paper, four types of features (*χ*, *ξ*, *ζ*, *α*) are used to find out the status of the lungs, that is, normal versus abnormal. These features are computed in different ways and discussed in the next.

Computation of kurtosis (*χ*) parameter: the kurtosis parameter describes the shape of the probability density function in terms of the flatness or peakedness [28]. The kurtosis value is greater for peaked distribution than that of the flat distribution. This parameter is the fourth order moment of the distribution and can be defined as

(9) $$\begin{array}{c} \text{Kurtosis}\left(\chi \right)=\frac{E\left[{\left(y\left(n\right)-\mu \right)}^{4}\right]}{{\left(E\left[{\left(y\left(n\right)-\mu \right)}^{2}\right]\right)}^{2}}-3=\frac{m^{4}}{\sigma^{4}}-3, \end{array}$$

where *E*(·) is the expectation operator, and *μ* = *E*[*y*(*n*)] and $\sigma =\sqrt{E[{\left(y(n)-\mu \right)}^{2}]}$ are the mean and standard deviation of the distribution, respectively, and *y*(*n*) is the *n*th sample value of the lung sound signal.

Estimation of skewness (*ξ*) parameter: the skewness parameter measures the asymmetry of the distribution [28]. A distribution will be asymmetric when probability density function extends unequally on the left or right sides of the center point. The skewness value is zero for symmetrical distribution and is positive or negative for asymmetrical distribution. The parameter is defined as the ratio of the third order moment and the cube of the standard deviation of the probability distribution and is calculated by

(10) $$\begin{array}{c} \text{Skewness}\left(\xi \right)=\frac{E\left[{\left(y\left(n\right)-\mu \right)}^{3}\right]}{{\left(E\left[{\left(y\left(n\right)-\mu \right)}^{2}\right]\right)}^{1.5}}=\frac{m^{3}}{\sigma^{3}}, \end{array}$$

where *m* ^3^ is the third order moment of the distribution and *E*(·) is the expectation operator.

Calculation of lacunarity (*ζ*) parameter: The concept of the lacunarity parameter was introduced by Mandelbrot to characterize the fractal objects [29]. This parameter can distinguish the objects with the same fractal dimension by measuring their texture information. The lacunarity feature was first implemented in the respiratory sound analysis by Hadjileontiadis to classify the adventitious lung sounds [30]. The following steps are involved in calculating the lacunarity value based on gliding box algorithm [26].

**Step 1**

Computate the box mass *b*_ma_ for a box of length *l* by placing it at the origin of the dataset of length *L* and *L* is always greater than *l*.

**Step 2**

Repeate Step 1 over the entire dataset by sliding the box with one space to the right direction.

**Step 3**

Calculate the probability distribution *P*(*b*_ma_, *l*) of the box masses by dividing the box masses *k*(*b*_ma_, *l*) by the total number of boxes *B*(*l*).

**Step 4**

Estimate of the first (*M*_1_) and second (*M*_2_) moments of the probability distribution.

**Step 5**

Calculate the lacunarity value for the size *l* by dividing the second moment by the square of the first moment and can be defined as

(11) $$\begin{array}{c} \zeta \left(l\right)=\frac{M_{2}}{M_{1}^{2}}, \end{array}$$

where *M*_1_ = ∑*b*_ma_ *P*(*b*_ma_, *l*) and *M*_2_ = ∑*b*_ma_^2^ *P*(*b*_ma_, *l*).

Computation of sample entropy (*α*) parameter: sample entropy (SampEnt) is a modified form of approximate entropy (ApEn) was introduced by Pincus. The SampEnt tool has been proposed by Richman and Moorman to reduce the bias caused by self-matching for each template of a data series [27]. It is defined as the negative logarithm of the conditional probability that two states that are match point-wise for a dimension *m* within a tolerance *r* remain match in dimension *m* + 1. The SampEnt calculation algorithm consists of the several steps that are described next.

**Step 1**

The templates or vectors of size *m* are formed from a given time series *v*(*n*)_*n*=1,2,…,*N*_ (in this case *v*(*n*) represents the lung sound signals) as follows:

(12) Um(j)=[v(j),v(j+1),…,v(j+m−1)],           1≦k≦N−m+1.

**Step 2**

The distance *d*[*U*_*m*_(*j*), *U*_*m*_(*i*)] between the templates *U*_*m*_(*j*) and *U*_*m*_(*i*) is defined as the absolute maximum difference of their corresponding scalar components and calculated by

(13) $$\begin{array}{c} d\left[U_{m}\left(j\right),U_{m}\left(i\right)\right]=\underset{k=0,1,\ldots ,m-1}{\max}\left(\left|v\left(j+k\right)-v\left(i+k\right)\right|\right). \end{array}$$

**Step 3**

Counting the number of templates matching for a given template *U*_*m*_(*j*) by considering the conditions: *d*[*U*_*m*_(*j*), *U*_*m*_(*i*)] ≤ *r* and *i* ≠ *j*.

**Step 4**

The conditional probability of template matching for a signal having *N* ^*m*^(*i*) number of templates matching for each template is computed as

(14) $$\begin{array}{c} C^{m}\left(r\right)=\frac{1}{N-m}\overset{N-m}{\underset{i=1}{\sum }}\frac{N^{m}\left(i\right)}{N-m-1}. \end{array}$$

**Step 5**

The sample entropy values are calculated by

(15) $$\begin{array}{c} \text{SampEnt}\left(m,r,N\right)=-\ln\left[\frac{C^{m+1}\left(r\right)}{C^{m}\left(r\right)}\right], \end{array}$$

where *C* ^*m*+1^(*r*) is the probability that two templates will match for *m* + 1 points.

## 4. Experimental Datasets and Implementation Issues

### 4.1. Subjects and Data Acquisition

The lung sound signals are recorded from the abnormal as well as normal male and female subjects with different types of pulmonary dysfunctions: Chronic Obstructive Pulmonary Diseases (COPDs), Interstitial Lung Diseases (ILDs) and asthma. These recordings are collected from various resources: Audio & Biosignal Processing laboratory, IIT Kharagpur and Institute of Pulmocare and Research, Kolkata, India. A total of 120 cycles are collected from 30 recordings of 10 normal and 20 abnormal individuals. The abnormal lung sounds include wheezes, crackles, and squawks sounds. The lung sounds are recorded from the anterior suprasternal notch positions of the subjects using a single channel data acquisition system and described in [22]. The sound recordings were performed in the sitting position and at relaxing mood of the patients, and stethoscope device was fixed tightly on the recording site to diminish the manmade artifacts. The acquired LS data were arranged in 16 bit, PCM, Mono audio format and stored as ∗.wav files at sampling frequency of 8 kHz. The recordings have been done with a large subjects of various age groups.

### 4.2. Implementation Platform

The whole analysis is implemented on an ACER-PC with 3.29 GHz Intel core 2 quad CPU and 3.49 GB of RAM. The MATLAB (R2008a, The Mathworks, Inc., Natick, MA) tool is used for conducting all the experiments.

## 5. Results and Discussion

The lung sounds data employed in this experiment are reported in Section 4. The datasets consist of pathological and normal respiratory sounds. These data are validated through three types of medical tests: chest X-ray, pulmonary function test, and high resolution computed tomographic (HRCT) scan. The decision regarding the lungs status, that is, normal versus abnormal, can be determined in an automated manner by using the machine learning properties. In this work, ELM and SVM networks are used to discriminate between normal and pathological individuals using their lung sounds characteristics. Both the classifiers use radial basis function (RBF) for processing the input sample data. The features are extracted from the 120 breathing cycles of 10 normal and 20 abnormal patients. The feature dataset is divided into five subsets: one subset for testing and the remaining four subsets for training. The training and testing processes are repeated five times for five individual feature subsets. The classification process runs through two phases, training and testing. During the training period, training dataset is rendered to classifier to form a generalized model that is used in examining the unseen data. In testing time, the unknown class feature set is verified by the trained model and it identifies the test data class. The performance of the classifier is evaluated in terms of percentage of classification accuracy (CA%), sensitivity (SEN%), and specificity (SPE%). The values of these measuring metrics are averaged on 5 trials of classification procedures for three different feature sets. These measuring units are defined by the following equations:

(16) $$\begin{array}{c} \text{CA}\left(\%\right)=\frac{\text{TP}+\text{TN}}{\left(\text{TN}+\text{TP}+\text{FN}+\text{FP}\right)}\times 100,\\ \text{SEN}\left(\%\right)=\frac{\text{TP}}{\left(\text{TP}+\text{FN}\right)}\times 100,\\ \text{SPE}\left(\%\right)=\frac{\text{TN}}{\left(\text{TN}+\text{FP}\right)}\times 100. \end{array}$$

The experimental results are shown in Tables 1 and 2. These results are obtained by employing the three sets of composite features vectors. Set 1 consists of two types of features (*χ*, *ξ*), set 2 comprises of three types of features (*χ*, *ξ*, *ζ*), and set 3 consists of four types of features (*χ*, *ξ*, *ζ*, *α*). Both the classifiers give better results for set 3 in comparison with the remaining two sets (i.e., set-1 and set-2) because of the proper modeling of respiratory sound using higher dimensional feature vector.  Table 1 shows that set 2 and set 3 give much better results than that of set 1, which means lacunarity and sample entropy features carry more relevant information than the skewness and kurtosis parameters. The training and testing time increase with the increasing feature dimensions. The SVM classifier takes more time than the ELM network. Unlike the SVM, the ELM network avoids the tuning of hidden layer biases and input weights. The ELM network gives slightly better performance than SVM due to its universal approximation capability of the target function. The efficiency of the proposed method is justified by comparing the experimental results with the baseline method introduced by Sello et al. [3]. The experimental results show that the developed method gives much improved accuracy over the baseline method and are shown in Table 2. The reason of poor performance of the existing method is that it only captures the energy information of the signal in terms of frequency quartiles features. These features cannot properly model the respiratory sounds. The quartiles features are extracted from the global wavelet spectrum, computed by the wavelet transform (WT). However, the efficiency of the WT depends on the selection of mother wavelet or basis function. The existing technique uses Morlet wavelet to generate the wavelet coefficients. On the other hand, the proposed method captures the structural information of the lung sound signals in terms of texture information, irregularity index, flatness, and asymmetric properties of the distribution. These features are inherently correlated with the morphological characteristics of the respiratory sounds and are capable of mapping the lungs conditions properly. Hence, the developed algorithm gives better performance than the existing technique.

## 6. Conclusion

This paper proposes a new method to detect the normal and abnormal conditions of the lungs in a non-invasive manner by exploring the inherent morphological characteristics of the lung sound signals using ELM network. The method is very fast because ELM network avoids the tuning of input weights and hidden layer biases by randomly selecting them during the learning process. The efficiency of the algorithm is tested for three combined feature vector sets. The proposed method gives a better accuracy of 92.86% than the baseline method which gives an accuracy of 87.66%. The proposed method is superior in terms of computational complexity and classification accuracy and can be used to develop an automated diagnostic tool that will assist the doctors in diagnosis the lung status: normal versus abnormal.

## Figures and Tables

**Algorithm 1:**
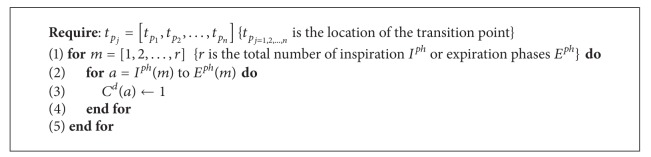
Calculate *C* ^*d*^.

**Table 1:** Performance of ELM and SVM classifier for different sets of the proposed feature vectors.

Type of classifier	Feature set	Feature vector	SEN (%)	SPE (%)	CA (%)	Training time (ms)	Testing time (ms)
ELM	Set-1	[*χ*, *ξ*]	52.94	69.38	62.82	0.75	0.57
SVM	Set-1	[*χ*, *ξ*]	61.56	59.71	60.71	6.50	2.10
ELM	Set-2	[*χ*, *ξ*, *ζ*]	85.71	87.50	90.47	0.83	0.65
SVM	Set-2	[*χ*, *ξ*, *ζ*]	85.71	77.38	85.71	17.00	5.70
ELM	Set-3	[*χ*, *ξ*, *ζ*, *α*]	86.30	86.90	92.86	4.20	3.70
SVM	Set-3	[*χ*, *ξ*, *ζ*, *α*]	86.30	85.70	91.60	23.60	8.10

*χ*: Kurtosis; *ξ*: Skewness; *ζ*: Lacunarity; *α*: Sample Entropy.

**Table 2:** Comparison of performances between the proposed and baseline methods [3].

Method	Feature vector	Type of classifier	SEN (%)	SPE (%)	CA (%)	Training time (ms)	Testing time (ms)
Proposed	[*χ*, *ξ*, *ζ*, *α*]	ELM	86.30	86.90	92.86	4.20	3.70
Method	[*χ*, *ξ*, *ζ*, *α*]	SVM	86.30	85.80	91.50	23.60	8.10
Baseline	[*f*_25%_, *f*_50%_, *f*_75%_]	ELM	80.47	79.71	87.66	2.62	1.10
Method [4]	[*f*_25%_, *f*_50%_, *f*_75%_]	SVM	90.90	72.60	86.33	10.70	7.75

SEN: sensitivity; SPE: specificity; CA: classification accuracy; *χ*: Kurtosis; *ξ*: Skewness; *ζ*: Lacunarity; *α*: sample entropy; *f*_25%_, *f*_50%_, *f*_75%_: quartiles features.
